# The Hospital. Nursing Mirror

**Published:** 1898-08-06

**Authors:** 


"The Hospital" Nursing Mirror.

Being the Nursing Section of "The Hospital."

[Contributions for this Section of "The Hospital" should be addressed to the Editor, The Hospital, 28 & 29, Southampton Street, Strand, London, W.C., and should have the word "Nursing" plainly written in left-hand top corner of the envelope.]

### News from the Nursing World.

### VICTORIA NURSES' HOME, GODALMING.

On the 26th ult. Lady Hilda Brodrick opened the Victoria Nurses' Home at Godalming. The home, which has been built in commemoration of Her Majesty's Diamond Jubilee, stands high, overlooking the town. The building is of red brick, is roofed with red tiles, and possesses more than one ornamental feature. On the ground floor is a hall, dining-room, matron's-room, ward-room, kitchen, and scullery. Upstairs there are four bedrooms, bath-room, lavatory, and above these, again, there is a large attic bedroom. The staircase leads direct from the hall. There are numerous cupboards, and the rooms are papered. Outside there is a coach-house for the accommodation of the ambulance van. On the occasion of the opening an awning was erected at the chief entrance, and gaily decorated. In spite of the rain a large company assembled to witness the ceremony, and the Mayor (Mr. Alderman T. Rea, J.P., C.C.), the Mayoress, Mr. and Mrs. Pullman, the Vicar (the Rev. L. H. Burrows), Miss Marshall, and Mr. W. H. Steggles received Lady Hilda Brodrick who was accompanied by Viscount Midleton, and the Hon. Arthur and Miss Muriel Brodrick. The new home has been built from the plans of Mr. S. Welman, of Godalming, at a cost of £878.

### IMPROVEMENTS AT THE WESTERN INFIRMARY, GLASGOW.

A new extension of the Western Infirmary, containing improved nurses' accommodation, has recently been opened at Glasgow. It is connected with the main buildings by means of a large well-stocked conservatory, and it consists of a large dining-room, a room for the servants who wait upon the nursing staff, a larder, and a service pantry. The dining-room has two large bay windows, and north headlights, and is fitted with electric lamps. The woodwork is stained green, and there is a pretty screen of the same tinted wood dividing the room from the corridor. It is furnished with a number of little tables, which are adorned by vases of flowers and plants. The china is pretty, and the meat is carved on a hot steel plate in the pantry and served as required. The new building is heated throughout with hot water radiators, and every convenience in the shape of lockers, hot and cold water, &c., has been provided.

### CHILDREN'S FLOWER FETE AT BARROW.

Very recently Mrs. Wadham, to whom the Barrow District Nursing Association owes so much, had the pleasure of opening a flower fête, in aid of the association, which had been arranged by the Children's Patriotic Society in aid of her own. The Town Hall was charmingly decorated for the purpose, efforts having been made to transform it into a garden. In addition to the handsome gifts of fruit and flowers by those either connected with, or interested in the societies, a large number of handsome palms and ferns were lent for the occasion. Mrs. Wadham was presented with a basket of roses by the youngest "patriot."

### A QUESTION OF REMUNERATION.

A question of considerable difficulty, involving several intricate points, was discussed lately by the Guardians of Enniskillen Union. In the early part of the superintendent of nurses' engagement she acted as superintendent to the staffs both on night and day duty, but, the work proving too arduous, a night superintendent was appointed at a salary of £25 a year. During a temporary vacancy the superintendent again undertook the night work, and at the end of it was granted £4 extra for the five weeks' work. The same thing has happened again, and the claim which she has sent in has been vigorously disputed, which dispute has ended in the nurse receiving £2 10s. It is well known that there are dozens of nurses who, in cases of emergency, put their shoulders to the wheel and do other work than that for which they are engaged and paid, and for which they neither ask nor expect extra remuneration. Sometimes extraordinary services are rewarded by an honorarium, but it is not well for this to be looked at in any other light. If there be work for two persons it is absolutely impossible that any one person can do it, especially if that work be of such an arduous and imperative nature as nursing. What often happens when one nurse in a staff falls out is that her work is distributed amongst her fellows, and perhaps the less important duties devolve on untrained persons, but where extra payment is to be made an extra person should be employed, and it seems to us obviously undesirable for the superintendent to send in a bill, however well she may have been able to do the work. Imagine the abuses that might creep in with such an "open door." A superintendent nurse is responsible for the good nursing of the institution over which she presides; it is her duty to meet emergencies and to provide against them. In order to do this she has a right to ask her committee to sanction necessary expenditure; not, we think, to undertake the extra work herself and then to send them in a bill.

### PARAMATTA CHILDREN'S CARNIVAL.

On June 10th a children's carnival was given in connection with the hospital ball. Nearly two hundred young people, in all the delight of fancy costume, assembled in the Town Hall, Paramatta, and enjoyed themselves thoroughly until half-past eleven p.m., when the festivities ended. The prizes for the best dresses were taken by Miss Lily Kemp and Mr. Bertie Harrison. Miss Kemp's costume represented Klondyke, and was of white satin trimmed with gold tinsel and fur; and Mr. Harrison's was that of Sir Charles Surface. The matron and nursing staff of the hospital decorated the supper-room, and their handiwork was much admired. The evening was a great success, and will add considerably to the funds of the hospital.

### CLERIC OR LAY NURSES.

The nuns have been the pioneer nurses of Rhodesia, and, now that the country is becoming more settled, the old question of employing cleric or lay nurses comes to the front. The root of the difficulty, of course, lies in what may be called the dual vocation. Nursing to the "Sisters" is an acceptable work with which to fill up the intervals of a life of prayer and devotion; while to the lay nurse nursing is the vocation, and prayer occupies the same relation to it as it does to any other secular occupation. It by no means follows that all the advantages lie on the side of the lay nurses. In Austria, for instance, they have been proved to be so much the reverse of what nurses ought to be that they are being replaced in certain institutions by nuns. This step is viewed by the professed lay nurses with so much distrust that they have founded a Nurses' Protection Society to uphold their interests. No society can protect the interests of the inferior workwoman. The thing to be borne in mind is that sound religious principle is the groundwork of a good nurse, be she cleric or lay, and the lay nurse cannot afford to forget it.

### NOTTS NURSING FEDERATION.

Lady Belper presided over the quarterly meeting of the Notts Nursing Federation, held in the Shire Hall, Nottingham, on July 27th. Mrs. Ball was asked to become lady superintendent, and to supervise the work and engagements of the staff nurse of the federation, and to make inquiries if some arrangement could not be made with the Hucknall Nurses' Home for boarding her when out of work. A sub-committee was appointed to select the uniform to be worn by the nurses engaged by the federation.

### A DIFFICULT QUESTION.

We have had occasion to refer more than once to the difficult question of the frangible nature of a contract or engagement, and the subject is illustrated by events occurring at the present moment at Bethnal Green. Nurse Allsop, the candidate appointed by the Board of Guardians, refused to take up the position, consequently she put them to the annoyance and expense of seeking another nurse. The Guardians have referred the matter to the Local Government Board, but not before an amendment was discussed as to the desirability of sueing the lady to recover a month's wages and damages. Two members of the board were of opinion that a nurse had the legal right to break her contract of engagement if she chose, and contended that under the present system of engagement she was perfectly free to accept another post until her appointment was formally notified. The decision of the Local Government Board in this matter must therefore be an important one to nurses generally. In our opinion, when a nurse accepts an appointment she ought to fulfil it, at any rate until such time as she can give proper notice to terminate her engagement. On the other hand, public bodies should act promptly in such things. A nurse cannot afford to wait two or three weeks on the chance of her election to a vacant billet, and is at perfect liberty to enter upon other work during negotiations, but not after appointment.

### AN EXCEPTIONAL NURSE.

It is frequently pointed out that one of the great drawbacks to the nurse's career is the early age at which she becomes incapacitated for her work. It is, therefore, a matter of congratulation when, as in the case of Nurse Whitbread, the worker reaches the limit of threescore and ten years by all but a year before she lays aside her task. This veteran nurse is 69 years of age, and for the last 15 years has been in the service of the Billericay Board of Guardians. She has just sent in her resignation and asked to be superannuated.

### SHORT ITEMS.

The railways of South Africa have granted the nurses of the colony the privilege of travelling at half fares.—The Government of New South Wales has refunded the money expended by the Bourke Hospital authorities in railway fares for nurses from Sydney during the late typhoid epidemic; it amounted to £40.—A bazaar, held in the rectory garden at Halesowen, in aid of the parish nurse fund, realised £15. Miss Bartlett has attended 161 cases during the last two years, and is popular with those she has nursed. There is a balance to the good in last year's accounts of £8 13s.—Wareham and Dorchester Boards of Guardians have both subscribed £2 2s. to the funds of the Dorset County Home for Nurses. They have the privilege of a nurse's services in case of need at £1 1s. a week.—On the 1st inst., Nurse Johnson, of Rochdale, takes up her work as parish nurse of Arundel in room of Nurse Skimmin. She is a member of the Royal British Nurses' Association.—The hon. secretary of the Queen Victoria Nursing Institution, Wolverhampton, has received a cheque for £25 from the executors of the late Right Hon. C. P. Villiers towards reducing the debt on the building of that institution.—Two memorials to her daughter, who died last year, have been established by Mrs. Munro Ferguson, of Novar, N.B. One is a bursary at St. Andrews University for lady students, and the other will be a nurse's home on her estate.—Lord Kelvin is the president of the Largs District Nursing scheme, of which the first year's report is published. It is a great success; the outlay has been £84, and the income £216.—At Nantwich the assistant nurse receives a salary of £30, and the superintendent £27 10s., with an allowance of £2 10s. for uniform. This anomalous state of things will soon rectify itself, as the superintendent's salary receives a yearly increase of £2 10s. —Lord Archibald Campbell opened a bazaar in aid of the funds of the Argyll District Nursing Association at the Jubilee Hall, Inverary, on the last Wednesday in July, which realized £230.—There are twenty nurses in course of training for plague duty at Bangalore city. Three have passed their examinations, and been placed on the reserve list, and are each drawing two rupees a month until their services are wanted.—Major-General Lee, J.P., who is the treasurer of the Barry Diamond Jubilee Fund, has received altogether £807 for it. The money will be devoted to building a nurses' home for that district.

### Antiseptics for Nurses.

By a Medical Woman.

### XII.—ANTISEPTICS DERIVED FROM SILVER SALTS, &c.

Silver nitrate has long been used as an antiseptic, and lately other silver compounds have been introduced which possess very active and efficient antiseptic and disinfectant properties. They have, moreover, been extensively tried on the Continent, and have in every case given very good results. From amongst these we will therefore describe the following.

Actol.—This is known chemically as a silver lactate. It is reported to be an excellent antiseptic, superior even to perchloride of mercury, and is one of the strongest and most effectual of the antiseptics derived from silver salts hitherto discovered. Actol is soluble in water, in the proportions of 1 part in 30 of water. Its use must, however, of necessity be somewhat restricted, as it is only capable of exerting its antiseptic properties when used as a solution. If used as a powder for dusting over wounds or raw surfaces, it causes the albumen to coagulate, and consequently the antiseptic cannot penetrate.

Itrol is a silver compound, known chemically as a silver citrate, and has been proved to be a good but not very strong antiseptic. It is only soluble in water in the proportion of 1 part in 4,000 of water. Itrol can, however, be used as a powder with satisfactory results to wounds.

Pure metallic silver has also been prepared in a form which is soluble in water that contains albumen. It is also made up as an ointment of the strength of three grains of silver in the ounce, and all these silver preparations have been reported as capable of exercising a "favourable influence on sepsis."

Argonin: As long ago as 1895 a German, named Rudolf Meyer, investigated with great care the bactericidal powers of this preparation of silver, and tested them as compared with those of silver nitrate and other disinfectants and found the comparison very favourable to argonin. Argonin is a compound containing 4 per cent. of silver combined with casein, and is soluble in water, with which it forms an opalescent fluid in diluted solutions, but when concentrated it is quite opaque. It becomes clear again, however, if a little ammonia or carbonate of soda be added, and the addition of the former, moreover, is a distinct advantage, as it increases the strength of the argonin as an antiseptic; but, on the other hand, by the addition of ammonia it is deprived of its bland and non irritating qualities, which make it so valuable as an antiseptic. Argonin is reported as being a fairly strong antiseptic, and when used alone in solution, without the addition of ammonia, it is neither corrosive nor irritating in its action upon the tissues of the human body.

Protargol is another preparation somewhat similar in character to argonin. It is a combination of silver with a definite protein, in the proportions of eight per cent. of the metal. It is a light yellow powder which is very readily soluble in water up to the strength of fifty per cent., and forms with water a perfectly clear, transparent light brown fluid. It is a powerful, efficacious antiseptic, and causes neither pain nor irritation when applied to wounds or raw surfaces, which is a remarkable property when it is taken into consideration what a large proportion of silver salts it contains, and that it possesses high powers as a bactericide. It is recommended as a spec'ally valuable application in the treatment of wounds and broken surfaces when there is much discharge.

Engol is a combination of several well-known but individually weak antiseptic substances, but each possessing certain recognised properties. It is thus another illustration of the principle which has been repeatedly proved by many careful bacteriological experiments that, under certain circumstances, if several of the comparatively weaker antiseptic substances be judiciously mixed together, a more effective and powerful antiseptic is thus produced, which may have the advantage over those which are individually stronger in being, as a rule, much less irritating to the tissues. In the case of engol there is a mixture of the following antiseptics, viz., boracic acid, eucalyptol, menthol, beta-napthol, and hamamelis, which are combined in certain proportions, and these form an almost colourless and transparent fluid, having a pleasant aromatic smell something like that of thymol. Engol is described as a very "powerful and agreeable antiseptic, and, moreover, is said to be non-poisonous." The statement, as to being non-poisonous must, however, be received with caution, since, as we have already pointed out, an antiseptic cannot be both powerful and not poisonous—that is, not poisonous if taken internally. The only way in which an antiseptic can be said to be non-poisonous is in the sense that it does not produce toxic effects if a reasonably strong solution is applied over a large raw surface.

Bicarbonate of soda, when chemically pure, has been used abroad as an antiseptic, and with marked success in cases of foul suppurating wounds and ulcers, to which it has been applied as a 2 per cent. solution by means of a compress. Bicarbonate of soda has long been used in weak solution in which to boil surgical instruments in order to sterilise them, and the bactericidal powers of a strong solution of washing soda are also well known.

Aristol is another combination of two weak antiseptics, viz., iodine and thymol. It is white in its solid form, but turns brown when exposed to the air. It is only very slightly soluble in water, but readily so in either alcohol or ether. It is recommended as a very efficient antiseptic.

Kersole is a mixture of various soluble tar products and salicylate of soda. It is very soluble in water, and makes an excellent antiseptic.

Phenosalyl is another mixed antiseptic composed of salicylic acid, phenol, menthol, eucalyptol, with benzoic and lactic acids, which are all heated together. It is a syrup-like clear fluid, is readily soluble in water, and is reported to have excellent antiseptic properties.

Phenol has also been combined with sulphuric acid, by which its action as a bactericide has been very much increased.

Orthoform owes its antiseptic properties to benzoic acid, and has been used in Germany. It is a fine white powder, very slightly soluble in water. Good results have been obtained by its use either as a powder or as an ointment. In addition to its antiseptic properties it is also a local anæsthetic, and consequently very effectual in cases of painful ulcers or burns.

Lozar is one of the most recent derivatives of phenol, and is prepared both as a liquid and a powder. It claims to be an "absolutely reliable and a most efficient disinfectant," and readily soluble in water. Its antiseptic properties have not yet been tested in surgery, but as it belongs to the phenol group of substances, it has good disinfectant properties.

Dermosol, an entirely different compound from dermattol, which has already been noticed, is one of the numerous products arising from the distillation of coal tar, being obtained as a heavy oily substance, which is next treated with a solution of potash. Dermosol is a milky-looking fluid which is soluble in alcohol and ether, but slightly so in glycerine. It is a mixture of cresol, creolin, benzol, terebene, &c., and is reported to be a strong microcide. It is best incorporated with vaseline or glycerine, when it loses its slightly irritating properties.

### Nursing in Paris Hospitals.

### C.—THE NURSING SISTERS.

### I.—HISTORICAL SKETCH.

TRULY the history of the Nursing Sisters of Paris is as much lost in the twilight of fable as that of the Roman Church, according to Macaulay. The oldest body is said to take its name from its founder, over twelve centuries ago, a certain Sister Saint Augustine, hence they are called Augustines. I am afraid the Sister Saint Augustine is something of a myth. The nursing nuns were originally under the auspices of the oldest of Christian monastic orders, the white friars and white nuns, who take their name from that great father of the Church to whom Christianity owes so much of its doctrine and its practice. In fact, the old name of the nursing nuns, "Hermit Daughters of Saint Augustine," indicates the reason of their name.

At all events, the premier nursing nuns are the children of the Hôtel Dieu; they took that ancient establishment as their "mother house," and they still retain it as their stronghold. The nursing white nuns of the Hôtel Dieu, besides various adherents in other French towns, spread ages ago into other countries—to Italy, Spain, the Netherlands, Germany, and even into England. Before the Reformation Saint Bartholomew's in London, among other hospitaliary institutions, was served by daughters of the Hôtel Dieu. In Paris itself, before the laicising campaign, the white Augustinians were found in three other hospitals beside their ancient home, that is at La Charité, Lariboisière, and where they still remain, at Saint Louis. Next in seniority to the Sisters of Saint Augustine come the "Daughters of Charity of St. Vincent de Paul," or grey nuns, commonly known as Sisters of Charity. Although not originally a Parisian order, they so early became domiciled here, and may be so said to have been refounded in Paris, that they may almost be as much counted as natives of the city as the Hôtel Dieu Sisters. Although instituted at La Bresse for nursing the sick peasantry, by Saint Vincent de Paul, among the numerous good works of his wonderful career, the order was introduced into Macon in 1623, and shortly after into Paris by the renowned Louise de Marillac (widow Legras), an enthusiastic disciple of de Paul, to which remarkable woman the Sisters of Charity owe their immense inspiration, success, and consequent renown. The headquarter of the Sisters of Charity has always been at the corner of the Rue de Bac and the Rue de Babylon, opposite the modern Bon Marché, and backing on the Laënnec Hospital in the Rue de Sèvres. It is also only a stone's throw from the immense enclosure of the Nuns of the Sacred Heart. The two neighbouring orders, each dating from the seventeenth century, seem to typify the antithesis of women's monasticism, that of merely extreme devotion for oneself and active devotional helpfulness towards others. The hospitals from which the Sisters of Charity were evicted in the late campaign were the Foundling Hospital, the Necker Hospital, and the Trousseau Hospital.

Following the Sisters of Saint Vincent de Paul in order of date come the Sisters of Saint Martha, an order established at the Hôtel Dieu of Perigeux, in 1643, for nursing the sick poor. Like the Saint Vincent order, the Saint Martha nuns were soon introduced into Paris, and Paris from that time became their chief seat. The Sisters of Saint Martha have played a peculiar part in the laic campaign. To understand their special and anomalous position one has to recall the ecclesiastical history of France and the quarrels of the Gallican Church, even to the great surrender to Rome in 1870, after the Church had ceased to have France at its back. The Saint Martha Sisters are fausenists children of Port Royal, and thus ecclesiastical pariahs of recent years, sternly ignored by the Roman hierarchy. I have referred to the dramatic episode in the Chamber of Deputies, when M. Constans sprang the information on his audience that the administration had been asked by the Sisters in certain hospitals to be relieved of their charge, these hospitals being served by the Sisters of Saint Martha. This important order, discarded by Rome and unsupported by the unbelieving multitudes, has dwindled away. All that were left of them eight years ago were in the three hospitals, La Pitié, Beaujon, and Saint Antoine, and when these were laicised the order ceased to exist. At La Pitié four of the Sisters enlisted as laic nurses, and one of the four I saw attending her duties only a few months ago.

Beside the three chief orders I have named, the 467 Matron-Sisters in the Paris hospitals in 1877 (the year before the great laicisation) included representatives of several minor and more modern orders, such as the Sisters of St. Joseph, a Parisian order of this century, whose chief function is teaching, but who had also the charge of the sick children's hospital. Another order was the Sisters of St. Mary, an order dating also from this century, which was installed at the Cochin Hospital.

Much stress has been often laid by the advocates of laicisation on the fact that the Hôtel Dieu records contain a series of charges of abuses among the Sisters extending over several centuries, and that several times have the Sisters been turned out *en bloc* and replaced by others; in fact, once, in 1505, as I have before remarked, by laics. This list of complaints against the Sisters dwindles into comparative nothingness, however, when we consider the length of time it represents, and the inevitable occasions of minor lapses from strict duty in such a vast number of changes of *personnel*. Moreover, even if the Matrons of the Hôtel Dieu have been changed on several occasions, the final result has been, as I have shown, for the original occupants to come back to their own.

It is a sufficient answer for the Hôtel Dieu Sisters to recall the fact that they outlived the anti-ecclesiastical fury of the Reign of Terror, and that even in the full blast of the Revolution the council of administration of hospitals reported that "the nurses of the Hôtel Dieu are mostly ancient nuns of this house. . . . For nobody is ignorant of what advantages the services of women are to the sick, above all when they are guided in their sad duties by superior views, which raise them above the aversions inspired by repulsive incidents of illness." Truly this tribute in the great hour of trial for the very basis of society far outweighs all the backstair scandals of the accounts of twelve centuries of service.

EDMUND R. SPEARMAN.

### Training in the Provinces.

(*Continued from page* 157.)

### KENT AND CANTERBURY HOSPITAL, CANTERBURY (106 Beds).

### TERMS OF TRAINING.

Candidates for training at the Kent and Canterbury Hospital should be between 22 and 34 years of age. Training is given under different circumstances for one, two, or three years, certificates being granted at the end of the second or third year. A few probationers are received for one year, paying a premium of £20, the committee reserving the power to return any portion of this sum should the probationer leave before the expiration of the year. These probationers have the option of remaining for a second year without further payment and without salary. Ten probationers are received for a term of two years' training, paying no premium, and receiving no salary. If retained for a third year they are given a salary of from £16 to £18 per annum. Uniform and laundry are provided by the hospital.

Promotion to the post of ward sister depends upon the character, education, and general capacity of the nurse. Preference is given to the special probationers, though sisters are sometimes selected from the other nurses if they possess the necessary qualifications. They are sometimes promoted at the end of two years; but if a sister is appointed who has trained entirely elsewhere a three years' certificate is essential. Sisters' salaries range from £25 to £30 per annum.

Night duty is taken by the nurses in their second and third years, these filling much the same position as "staff nurses" in some hospitals. They take three consecutive months of night work during the twelve months.

### HOURS ON AND OFF DUTY.

The working hours number about nine out of the twenty-four on an average. Probationers go on duty in the wards in the morning at 7, leaving them for the night at 8.30 p.m. Out of these hours must be deducted one hour and three-quarters for meals, and an hour and a half to two hours and three-quarters for recreation. This is taken on alternate days from 12 to 1.30 p.m., and from 3 to 5.45 p.m. One whole day off is given in the month, and four weeks' annual leave, one week being taken after the first six months' work. On their days off probationers do not enter the wards at all, and may, if they choose, spend the day in bed, having their meals sent to them there.

Sisters go on duty in the morning at 8 o'clock, and leave their wards at 9.15 p.m. They are off duty alternately in the afternoon and evening, as the state of their ward permits, and every other Saturday they have leave of absence from 2 p.m. to 9 a.m. the following day. They have also one whole day off each month, and one month's annual holiday.

Night nurses are on duty from 9 p.m. to 8.30 a.m. It is a rule that at least seven hours be spent in bed.

### MEALS.

The day staff breakfast at 6.30 a.m.; luncheon is from 9 to 9.30; dinner 1.30 to 2 p.m.; tea 5 to 5.45 p.m.; supper at 8.45. The only meals not served in the dining-room are the night nurses' midnight ones. These are taken in the ward kitchens. All other meals are laid in the dining hall of the nurses' home. Certain allowances are made per head per week, though this is not adhered to with very great rigidity, but no stores are given into the nurses' hands.

Sisters' meals are as follows: Breakfast, 7.30 a.m.; dinner, 1 p.m.; tea, 4.30 to 5 p.m.; supper, 9 p.m.

### ADDENBROOKE'S HOSPITAL, CAMBRIDGE (158 Beds).

### TERMS OF TRAINING.

The terms upon which probationers are received at Addenbrooke's Hospital are somewhat unusual. No probationers are received without payment, the fees being £10 10s. a quarter for the first year and £5 5s. a quarter during the second year. For the third year no payment is required, and no salary given. The probationers are not bound by any agreement, but no certificate is given until the completion of the third year of training. All probationers are expected to provide their own uniform. The probationers in their third year are promoted to take staff duty under the sister of the ward. Lectures are given to the nurses during training by members of the medical staff.

Any probationer who possesses the necessary qualifications is eligible for promotion to the post of sister when her training is completed, but further experience elsewhere is considered very desirable, preference being given to Addenbrooke's nurses when a sister's appointment is vacant. Such vacancies rarely occur. Sisters' salaries begin at £30 per annum, rising to £35 after three years' service, with £5 for uniform.

The accommodation for the nurses is extremely comfortable, separate rooms being provided for all in the home, and that very important adjunct to every nursing home, a quiet and restful "sick-room" is a special feature at "Addenbrooke's."

The night nursing is done by probationers in their second and third years, under the superintendence of a permanent night sister, day and night duty being taken in alternation for periods of three months.

### HOURS ON AND OFF DUTY.

Probationers go on duty at 7 a.m., and leave their wards in the evening at 8.30. From these hours must be deducted half an hour each for lunch, dinner, and tea, and two hours to two hours and three-quarters daily for recreation. The average hours actually spent in the wards therefore amount to about nine and a quarter. There are no definitely fixed days off, but they are given now and again as it can be arranged. On Sundays practically the nurses have half-days, either in the morning or the afternoon, and if the former it is often arranged for them to have breakfast in their own rooms in order to secure for them as much rest as possible. The sisters' hours off duty are not fixed; they are arranged for as may be found convenient. It should be mentioned that in a hospital where, as at Addenbrooke's, the medical staff pay their visits in the morning, the afternoon is a very slack time, unless there may be any specially heavy cases in the wards, and that therefore in practice the sisters are able to obtain sufficient time for recreation without any difficulty. One month's holiday is allowed yearly.

### MEALS.

The day nurses breakfast at 6.30; from 10.15 to 11.15 lunch is on the table in the dining-hall for the probationers to go to as may be convenient, half an hour being allowed for the meal. At 1.30 the matron dines with the sisters and some of the nurses, the second dinner following at 2.15. Tea is in the dining-hall from 5 to 6 o'clock, and supper is at 8.30. No meals, except the night nurses' midnight meal, is served out of the dining-hall. The night nurses take their provision for the night with them from their breakfast each evening. The weekly allowances of tea, &c., are kept in special lockers in the dining-hall.

### A Book and its Story.

"GLADSTONE: THE MAN."

"While other pens have been busy recording and criticising the political acts of Mr. Gladstone, mine will be the more grateful task," writes the author of this little work* on the great statesman, "of portraying him, not as a politician, not as the statesman who guided the helm of the ship of State during four eventful Premierships, but as the man whose personal character is esteemed by political friend and foe alike." And all who read Mr. Williamson's book will readily agree that he has succeeded in a very admirable manner in the task he has set himself of making us better acquainted with the personal side of the career of one of the great men of our day.

Of Mr. Gladstone's early career there is not much recorded which cannot be briefly summarised. His father was Mr. John Gladstone, a prosperous merchant at Liverpool, who, at the suggestion of Sir R. Peel, was created a baronet in 1845. He was married twice. His second wife, Miss Robertson, daughter of a Provost of Dingwall, was a lady of great accomplishments, of fascinating manners, and of high intellect. She was the mother of William Ewart Gladstone, who was born in Rodney Street, December 29th, 1809. The house in Rodney Street was destined, curiously enough, to be the home of two successive politicians, for the son of Mr. John Cardwell, who succeeded in the tenancy of the Gladstone's home, became eventually a Cabinet Minister and a member of the British Peerage, under the title of Viscount Cardwell.

Three months before he attained his twelfth birthday the young William Gladstone was sent to Eton—"that queen of visible homes for the ideal schoolboy," as he described the place in after years. Here he began his long list of literary productions, and here, too, he entered with all the zeal of his youthful years into the debating society of the school. In 1827, "the prettiest little boy that ever went to Eton," as Sir Roderick Murchison called him, had developed into a slim, scholarly young man. "Leaving the famous school, where his name carved in the oak by himself is an object of veneration, and where recently a bust was placed to his honour, as the result of subscriptions from members of Parliament of all shades of politics," he continued his education under Dr. Turner; then he entered Christ Church, Oxford, where, in 1831, he took a double first class and held for a short time a Fellowship at All Souls' College; even in those youthful years it was a foregone conclusion on the part of his contemporaries that he would come to be a leader of men, for which no one was better fitted than was young Gladstone, who brought the strength of sustained purpose to bear on a mental grasp of no ordinary power. Mr. Gladstone's love for his Alma Mater, which he has described as "the fountain of blessings, spiritual, social, and intellectual," an admiration commenced in his undergraduate days, remained with him throughout life, and the first efforts of a literary career, which was destined to be a fruitful one, were dedicated to the University of Oxford in his work on "The State in its Relation to the Church," published in the autumn of 1838. Thus was the foundation-stone laid of that monument of literary activity to which Mr. Gladstone was adding almost every year of his life. It would be impossible, says Mr. Williamson, "to compile a complete Gladstone bibliography, for the mere enumeration of Mr. Gladstone's publications fill a great space in the British Museum catalogue." Later on his biographer remarks, "It is fitting that the last volumes which bear his name should be those connected with theology, just as his earliest literary successes were gained in the same field."

Mr. Gladstone's home life is charmingly described by Mr. Williamson, and we would refer the reader to such chapters as deal with the family circle at Hawarden, and to the excellent illustrations which accompany them of the Castle and its interior and groups of all who formed the home party there. In any record of Mr. Gladstone's life, an important place must be allotted to his marriage, "for his long and happy union had much to do with the peace of mind which enabled him to accomplish so much public work. 'Marriage is promotion,' says a distinguished writer, and certainly Mr. Gladstone's was promotion in the best sense." His wife was the daughter of Sir Stephen Glynne, of Hawarden Castle, in Flintshire; after her marriage the estates passed to Mrs. Gladstone. Mr. Williamson confines his readers' attention almost entirely to the private life of Mr. Gladstone, and it is not as the politician, with whom all may not be in sympathy, that he describes the subject of his biography, but it is as "Gladstone: the Man" that he writes, and all who have minds—minds with which to comprehend and hearts with which to feel—will be conscious of a very reverential sympathy with a man whose purposes were so high, whose aims so lofty, and who, to the finish of a long lifetime, carried with him the beautiful childlike faith which characterised his varied actions. Of this side of the statesman's career, whether we be in political sympathy with him or not, it is good for us all to read, and many a lesson may be learnt by the contemplation of such a life. We have said Mr. Gladstone lived a long life, longer, indeed, than is usually allotted to man to live, but he never grew old—that is, not old in the sense in which that term is so often read; his vital interest in mankind and things around him kept him young. And his interest in all that was going on in the world in which he lived was unabated to the end; if one could learn no other lesson in William Ewart Gladstone's life this at least we all can learn, and it is no mean a lesson after all if we think over the full significance of what it means—the duty of keeping the mind young and ready to the end, to be invoked in such activity as is beneficial to our fellow creatures.

Among his many qualifications, as writer, orator, and omnivorous reader, Mr. Williamson speaks at some length in eulogy of Mr. Gladstone's powerful oratory, for as an orator Mr. Gladstone stood alone. Short or long—and they were often very long—his speeches were always worth hearing, and in them were "always some purple patches" of real eloquence. When he spoke in the library of the National Liberal Club and made an appeal for volumes to fill its shelves, there was apparent the bookman's zeal; when he pleaded for the cause of charity, sincere sympathy with suffering was evident; when he responded at the Mansion House for Her Majesty's Ministers, there was the strong sense of responsibility. Behind all the speeches, whenever and wherever delivered, there shone the bright light of a great personality, and the radiance of the light was never dimmed even in Mr. Gladstone's last days:—

"Great! for he spoke and the people heard,
And his eloquence caught like a flame
From year to year of the world, till his word
Had won him a noble name."

The last chapter but one in Mr. Williamson's fascinating little account of "Gladstone: the Man," is headed "An Appreciation," and among other tributes here paid to the memory of the great man is his courtesy, "so particularly charming and so freely accorded to all who met him." The writer declares that if all the volumes of biography published during the last half century were searched not a single instance of Mr. Gladstone's discourtesy could be found, even in the face of virulent denunciation and unmanly rudeness on the part of others; also, adds his biographer, there is hardly a case of an unkind phrase in his reputed utterances. The last scenes in the life of the departed statesman are all too freshly stamped upon our memories to need repetition here; all differences, political and otherwise, were laid aside, and the nation mourned with common voice the end of a great career, when, on Ascension Day, William Ewart Gladstone passed away after 88 years of strenuous life spent in the service of his countrymen.

* "Gladstone: The Man." A Non-Political Biography. By David Williamson. (London: James Bowden. 1898. Price 1s.)

### Everybody's Opinion.

[Correspondence on all subjects is invited, but we cannot in any way be responsible for the opinions expressed by our correspondents. No communication can be entertained if the name and address of the correspondent is not given, as a guarantee of good faith but not necessarily for publication, or unless one side of the paper only is written on.]

### LADIES AND GENTLEWOMEN.

"Matron" writes: I have on several occasions advertised for probationers in your journal, giving no definite qualification for the post except "gentlewoman," and in reply I receive numerous applications from all sorts and classes. Of course, everyone is a lady, but it seems to me highly desirable that the term "gentlewoman" should be recognised as necessarily implying good birth and education. Much useless correspondence would thereby be avoided, for, in my opinion, all candidates should in courtesy receive a reply to their application. I am writing in the hope that you may find space for this letter in The Hospital, and that it may induce some who are seeking probationers' posts to reconsider hasty applications.

### BOARD SCHOOL NURSES.

Miss Honnor Morten writes: May I briefly answer in one phrase both your leader of July 23rd and Miss Wardell's letter of July 30th? It is necessary that the school nurse should be thoroughly trained in order that she may not trench on the doctor's province. Much of her work will be preventive, and will consist in seeing when a child should be sent to a doctor, and so stopping these terrible outbreaks of diphtheria and measles which empty our schools. We believe the work to be so important that only the best nurses are fit for it. May I also state that the first nurse appointed is Miss Rose F. Petty, who was trained for three years at the London Hospital, and besides their certificate holds the Sanitary Institute and South Kensington advanced hygiene certificates. She has also been a teacher, and has seen much of children educationally and from the nursing point of view.

*** We agree. The more likely a nurse's work is to trench upon the medical the more fully trained should be the nurse who is employed. The well-trained nurse is far less likely to overstep the limits of her duty than one who has that fatal thing, "a little knowledge."—Ed. *T. H.*

### Minor Appointments.

Walmersley House Hospital for Incurables, near Bury.—On July 22nd, 1898, Miss Isabel Mackenzie Macdonald was appointed Matron of this hospital. She was trained at Longmore Hospital, Edinburgh, and was later night superintendent of the City Hospital, Edinburgh. She has been sister for five years at the institution of which she has just been appointed matron.

Reigate and Redhill Cottage Hospital.—Miss Lucy Violet Callender was appointed Matron of this hospital on July 27th, 1898. She was trained at the Hospital for Sick Children, Great Ormond Street, and at the Middlesex Hospital, where she was afterwards sister of the Princess May Ward from 1894-5, and of the Founder and Pepys Wards from 1895-8.

St. Olave's Union Infirmary, Rotherhithe, S.E.—On July 27th, 1898, Miss Alice Elizabeth Little, who was trained at St. Mary's Hospital, Paddington, was appointed Assistant Matron of this infirmary. She was ward sister Paddington Infirmary from 1894-6, night superintendent St. Saviour's Union from 1896-8, and assistant matron Kent County Asylum from that date.

Bradford Workhouse Hospitals.—Miss Mary Griffiths was appointed Night Superintendent of these institutions on July 27th, 1898. She holds a three-year certificate from Brownlow Hill Infirmary, Liverpool, and since obtaining it she has been charge nurse at the General Infirmary, Aylesbury, Bucks, and at the Union Infirmary, Leeds.

Poplar and Stepney Sick Asylum, Bromley, Middlesex.—Miss Maud Rodgers, who was trained at the Union Infirmary, Birmingham, has been appointed Sister of this asylum. She has been staff nurse at the Hospital for Diseases of the Heart, Soho Square, W.

### Death in Our Ranks.

The Grosvenor Hospital for Women, in Vincent Square, Westminster, has sustained a sad loss by the death of its Lady Superintendent, Miss Katherine Hughes. Appointed just twenty years ago, when the hospital was a very humble affair indeed, Miss Hughes has watched over its development with unremitting care and attention, and has so thoroughly identified herself with its work and progress that it is difficult for those who knew her well to imagine how the hospital can continue its career without her. During those twenty years the changes in the *personnel* of medical and nursing staffs, general and ladies' committees, have been numerous, and the very buildings have been entirely demolished and rebuilt; but the superintendent was generally regarded as a connecting link to bind these varying elements together and secure continuity of action and practice. Possessing in an eminent degree the qualities so necessary to her position, of good temper, tact, and the most unselfish regard for the comfort and well-being of all under her charge, Katherine Hughes made firm friends of all her patients, and endeared herself to her subordinates to an extent which is, perhaps, only now realised in the bitterness of their sorrow. Miss Hughes's professional training was carried out, we believe, first in an Irish hospital, then at University College Hospital, and finally at "The London," of which she ever spoke with affectionate regard.

### Medical Women and Nursing Sisters.

### NEW RULES FOR THE PLAGUE NURSES IN BOMBAY.

We have received from a sister on plague duty a copy of the following rules, which appeared in the *Bombay Gazette*:—

I.—The lady doctor is responsible to the medical officer of health and to the chairman of the municipality for the good conduct of the establishment, and it is trusted that the sisters will cordially support her.

II.—Sisters wishing to communicate with the authorities must do so through the lady doctor.

III.—Sisters must be in to dinner every night, and must not leave the premises after dinner except with the leave of the lady doctor. Such leave will not be granted more than once a week, and then only till eleven p.m., except under very special circumstances; the address at which the sister will be must be given to the lady doctor.

IV.—Sisters going out driving are requested to let the lady doctor know their destination and their escort (if any), and she is given authority to modify the arrangements made if she deem it necessary.

V.—Visitors to the sisters must send in their card to the lady doctor.

VI.—Sisters to be in bed by eleven p.m.

VII.—In case of sickness the lady doctor is to be at once informed, and she shall, if she deem it necessary, or if the sister shall desire it, call in one of the senior medical practitioners of Calcutta; she shall also make such arrangements for the invalid as shall appear necessary, with the advice of the medical attendant, subject to the approval of the medical officer of health.

VIII.—The household expenses are to be provided for by a fixed contribution from each person to be made as soon as the monthly salaries are paid. The payment to be made through the lady doctor.

IX.—At present each person is to contribute 110 rupees a month for board, lodging, light, and fuel, and the use of the servants provided by the landlady.

X.—Personal servants, punkah wallahs, ice, wine, and aërated waters, dhobie and ghari-hire to be provided by each person for herself.

XI.—There must be at least one personal servant between every two or three sisters; some public duty may be required from such servants at the discretion of the lady doctor.

XII.—No orders to be given by the sisters to any except their own personal servants and punkah wallahs, except through the lady doctor.

XIII.—Any complaints about housekeeping to be made through the lady doctor.

XIV.—Visitors may be invited to afternoon tea with the consent of the lady doctor. Ladies may occasionally be invited to other meals (with the consent of the lady doctor), at the expense of the person who invites them.

XV.—The rooms to be allotted at the discretion of the lady doctor, who may subsequently alter the arrangements if she shall consider it advisable.

### Notes and Queries.

**The contents of the Editor's Letter-box have now reached such unwieldy proportions that it has become necessary to establish a hard and fast rule regarding Answers to Correspondents. In future, all questions requiring replies will continue to be answered in this column without any fee. If an answer is required by letter, a fee of half-a-crown must be enclosed with the note containing the enquiry. We are always pleased to help our numerous correspondents to the fullest extent, and we can trust them to sympathise in the overwhelming amount of writing which makes the new rules a necessity.**

**Every communication must be accompanied by the writer's name and address, otherwise it will receive no attention.**

*Probation in Children's Hospitals.*

(173) I am desirous of obtaining a place as probationer in a children's hospital, preferably in London. I should require a salary. My age is 21½. Can you give me the names of any hospitals where I can apply?—*L. M. W.*

Both the East London Hospital for Children, Shadwell, E., or the Evelina Hospital for Sick Children, Southwark Bridge Road, S.E., and others, fulfil your requirements.

*Young Probationers.*

(174) Kindly tell me if there is any hospital in Staffordshire that is a recognized training school (if possible, near Walsall) where probationers receive a small salary from commencement, as I am anxious to train as a nurse. Age 21½ years.—*An Inquirer.*

You are too young yet for a general hospital. Why not apply to the matron of the Rugeley District Hospital? Although this institution grants no certificates, yet the experience would be useful, and the salary for the first year is £10.

*Private Staffs in India.*

(175) Kindly tell me if there are private nursing staffs connected with any of the larger hospitals in India? If so, would a nurse with a three years' certificate and the L.O.S. degree be likely to be received on one?—*R. O. S.*

We cannot give you any information respecting private nursing staffs in India. With your certificates and training you would probably succeed well after you had once formed a connection. In "Burdett's Hospitals and Charities" you will find a complete list of the hospitals of India, with the name of superintendent of nurses. Choose those most likely to suit you, and write to the superintendents for advice.

*Ingham Infirmary.*

(176) Kindly let me know if you consider Ingham Infirmary, South Shields, a good training school.—*Nurse Mona.*

The training is only for one year and in nursing accidents, you must, at the end of the year, seek a further two years' training in a general hospital, and make arrangements beforehand to have a combined certificate for three years from your two training schools.

*Xeroform.*

(177) Kindly tell me where I might obtain the powder mentioned as xeroform in THE HOSPITAL for June 4th, page 172.—*L. L.*

Xeroform can be obtained and is supplied by Burgoyne, Burbidges, and Co., 12, Coleman Street, E.C., and manufactured by Von Heyden's Chemical Co., Radebeul, Dresden.

*Mentone.*

(178) Winter nursing in Mentone. Address wanted of an institution.—*Nurse.*

(179) I should be glad if you could give me any information about the Holland House Institute, or could send me Miss Woodcock's address. I hear she takes nurses to the Riviera for the winter, and I should like to know how to put myself in communication with her.—*Trained Nurse.*

The Holland House Institute for English Trained Nurses has a branch at Mentone. Address the Director, 1, Tavistock Chambers, Bloomsbury.

*Dispensing.*

(180) What would you consider the best way to qualify for the dispensing examination of the Apothecaries' Hall, and what would be the fees?—*District Nurse.*

See "How to Become a Dispenser," "Mirror," April 3rd, 1897. The fee for the Apothecaries' Hall examination is £2 2s.

### For Reading to the Sick.

"AMONG whom ye shine as lights in the world."—*Phil.* ii. 15.

**Verses.**

Walk in the light! so shalt thou know
That fellowship of love
His spirit only can bestow,
Who reigns in light above.

Walk in the light! and sin abhorred
Shall ne'er defile again;
The blood of Jesus Christ the Lord
Shall cleanse from every stain.

Walk in the light! and thou shalt see
Thy path, though thorny, bright;
For God by grace shall dwell in thee,
And God Himself is light.

—*Zoriah Bateman.*

His high endeavours are an inward light
That makes the path before him always bright.

—*Wordsworth.*

How shall a child of God fulfil
His vow to cleanse his soul from ill,
And raise on high his baptism-light,
Like Aaron's seed on vestment white
And holy-hearted Nazarite.

First, let him shun the haunts of vice,
Sin feast, or heathen sacrifice;
Leaving the board of wealthy pride
Or heretic, self-trusting guide
Where the adulterer's smiles preside.

Next, as he treads the maze of men,
Aye must he lift his witness, when
A sin is spoke in heaven's dread face,
And none at hand of higher grace,
The cross to carry in his place. —*Newman.*

**Reading.**

"Let your light so shine before men that they may see your good works and glorify your Father which is in heaven."—*Matt.* v. 16.

"Truly the light is sweet."—*Ecclesiastes* xi. 7.

"Ye are all the children of the Light and the children of the day."

We may be on a sick bed; we may be in a darkened chamber; we may be but a dying frame; and yet we may shine as lights in the world, just as John Baptist was a burning and a shining light though "shut up in prison"; and as St. Stephen's face shone like "the face of an angel" amidst the stone-storm.

Shine amidst the worldly. They seem to have everything that heart can wish. All is gaiety, flattery, flutter, and a "vain show." You may be poor and sick, friendless and alone. Yet they may be wrong, you right. Light shines on the worldly, that makes the glitter. Light shines from the godly, that fulfils the command.

Shine amidst the unstable. When faith halts, love flickers. Many shine for a time, then their light goes out. They wander from place to place. They are drawn aside by every new thing. They are blown hither and thither by "every wind of doctrine." Keep out of the draughts, oh, Christian, and your light will so shine before men, they will see your good works and glorify your Father in heaven.

### Wants and Workers.

NURSE EMILY HAMBLIN, 53, Cadogan Street, Cadogan Square, S.W., offers several years' unbound copies of THE HOSPITAL to any nurse sending for them and paying carriage.

